# Hysterosalpingography with Oil-Soluble Contrast Medium Does Not Increase Newborn Hypothyroidism

**DOI:** 10.1155/2022/4532714

**Published:** 2022-02-22

**Authors:** Divya M. Mathews, Jane M. Peart, Neil P. Johnson, Robert G. Sim, Natasha L. Heather, Dianne Webster, Susannah O'Sullivan, Paul L. Hofman

**Affiliations:** ^1^Liggins Institute, University of Auckland, Auckland, New Zealand; ^2^Paediatric Endocrinology, Starship Children's Hospital, Auckland, New Zealand; ^3^Auckland Radiology Group, Auckland, New Zealand; ^4^Repromed Auckland, Auckland, New Zealand; ^5^University of Auckland, Auckland, New Zealand; ^6^Robinson Research Institute, University of Adelaide, Adelaide, Australia; ^7^Newborn Metabolic Screening Programme, LabPlus, Auckland District Health Board, Auckland, New Zealand; ^8^Endocrinology, Greenlane Clinical Centre, Auckland District Health Board, Auckland, New Zealand

## Abstract

**Objective:**

Hysterosalpingography (HSG) with oil-soluble contrast medium (OSCM) improves pregnancy rates in women with idiopathic infertility. However, OSCM has high iodine content and slow clearance resulting in potential iodine excess. If pregnancy occurs, this could impact fetal thyroid gland development and function. We aim to determine the effect of a preconceptional OSCM HSG on the thyroid function of the neonate. *Design and Patients*. This was a retrospective analysis of newborn TSH data for a cohort of neonates conceived within six months of an OSCM HSG in the Auckland region, New Zealand, from the years 2000 to 2019. Thyroid-stimulating hormone (TSH) levels of these newborns were obtained from newborn screening, which is routinely performed for all children at 48–72 hours of life. The primary outcome was the incidence of permanent or transient congenital hypothyroidism in this cohort.

**Results:**

Of 146 babies included, all had normal TSH levels with values ranging from 1 to 7 mIU/L on the whole blood analysis of a capillary heel sample using the Perkin–Elmer AutoDelfia assay. Conception during the first 3 cycles following an OSCM HSG was 76%; however, TSH levels in this group were not higher than those conceived in later cycles.

**Conclusion:**

Preconceptional OSCM HSG did not increase the risk of congenital hypothyroidism in the New Zealand scenario.

## 1. Introduction

Congenital hypothyroidism (CH) is a preventable cause of intellectual disability. Routine newborn screening is recommended [1] and widely employed by most countries because it enables early detection of CH and initiation of thyroid hormone replacement therapy and thereby perseveration of normal intellectual and cognitive function [1–3]. Although the most common causes of congenital hypothyroidism are dysgenesis and dyshormonogenesis [4], maternal iodine excess can cause prolonged primary hypothyroidism in the offspring [5]. The recommended daily allowance (RDA) for iodine intake established by the Australian National Health and Medical Research Council (NHMRC) is 150 *μ*g/day in adults and 220 *μ*g/day in pregnant women [6]. The likely mechanism of thyroid dysfunction with iodine excess is fetal iodine exposure and a prolonged Wolff–Chaikoff effect causing suppression of fetal thyroid hormone release [7]. Newborn thyroid function is well described with maternal iodine excess, particularly with the use of amiodarone (75000 *μ*g iodine/tablet) in the last trimester of pregnancy [8–12], faulty formulations of prenatal supplements, and dietary iodine excess intake or topical iodine [13–17].

Hysterosalpingography (HSG) using oil-soluble contrast medium (OSCM) is increasingly employed in infertile women as they improve fertility with at least a doubling of pregnancy rates when compared to no intervention [18, 19] and higher rates when compared to water-soluble contrast media (WSCM) (39.7% vs. 29.1%, respectively, in the H2Oil study) [20]. OSCM has a very high iodine content (480 mg iodine/ml of Lipiodol (Guerbet, France) and slow clearance with a half-life of 50 days [21]. Consequently, OSCM HSG is known to cause a 100-fold rise in iodine levels which fall slowly over the next six months to one year [22]. The fertility enhancement from OSCM is also maximal in the initial six months following the HSG [23]. Thus, the fetus conceived is at a risk of severe iodine exposure and subsequent problems with thyroid gland function.

Thyroid safety of children conceived following OSCM HSG in the context of potential iodine excess remains unclear [24]. A Japanese study suggested an increased incidence of newborn thyroid dysfunction in offspring conceived immediately following an OSCM HSG [25]. The incidence of thyroid-stimulating hormone (TSH) elevation was 2.4% (5/212 screened) and overt hypothyroidism was 0.94% (2/212 screened) as against 0.07% [26], the background rate of congenital hypothyroidism in the Japanese population. Interestingly, two of the five neonates with thyroid dysfunction had >20 ml OSCM (compared to a standard technique of <10 ml [27]) instilled in their mothers during the HSG and in three of the cases, the HSG to conception gap was less than two months suggesting that greater iodine exposure could be a risk factor for developing neonatal thyroid dysfunction. However, this pattern of increased thyroid dysfunction in newborns was not replicated in a later study in the Netherlands [28] or China [29].

Our research was conducted to determine the effect of preconceptional OSCM HSG on the newborn's thyroid function using the standard radiological practices followed in New Zealand [27].

## 2. Methods

We conducted a retrospective cohort analysis of newborns conceived following OSCM HSG in the Auckland region, New Zealand, from 2000 to 2019. TSH data from the newborn metabolic screening programme (NMSP) were extracted and used for the analysis. The cohort was identified from the database of OSCM HSGs kept by a fertility specialist and the radiology practice performing these procedures in the region from the year 2000 onwards. The standard approach for OSCM HSG in Auckland, New Zealand, involved using one ampoule or less of Lipiodol (usually 4–10 ml Lipiodol) during the HSG, which was performed in the follicular phase (between 5^th^ and12^th^ day of the cycle) [27].

**Figure 1:**
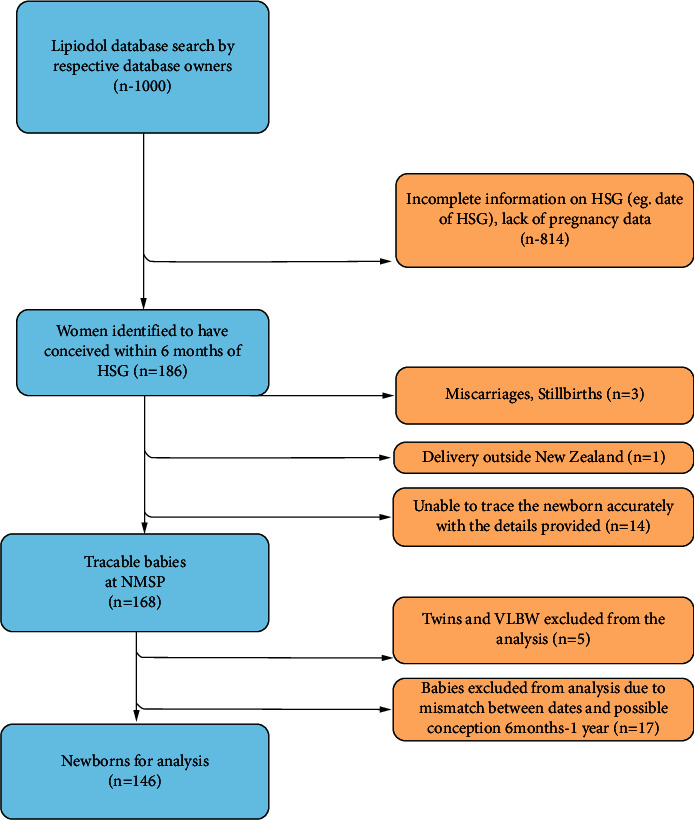
Flowchart for the identification of newborns conceived following OSCM HSG and the neonates included in the analysis of TSH data. HSG, hysterosalpingogram; NMSP, newborn metabolic screening program; VLBW, very low birth weight.

The women who conceived within six months of OSCM HSG were identified, and the TSH information of these babies was obtained from the NMSP. Necessary ethical approvals were obtained for this retrospective study. Confidentiality was maintained by ensuring that only coded data was received by the researchers from the custodians of databases and NMSP. If a child required referral to a paediatric endocrinologist for further assessment or initiation of thyroid hormone therapy, this was also recorded. The flowchart for cohort identification is shown in Figure 1. We included the babies who were confirmed as conceived within six months of HSG (based on the date of HSG, date of delivery, and gestational age) and excluded those who were very low birth weight (VLBW), i.e., those less than 1500 grams at birth, multiple births, or where newborn screening data were not available.

The baseline characteristics of the newborns identified by the NMSP as OSCM offspring (*n* = 168) are described along with demographics of all babies screened by the NMSP (population comparison) in Table 1. 146 babies were included in the study after exclusion as described in the flowchart in Figure 1.

New Zealand introduced newborn screening for CH in 1981 and adopted a TSH‐only screen in 1986. Newborn dried blood spot (DBS) screening employs capillary heel blood collected onto a card and is offered routinely for all children. Most samples are collected between 48 and 72 hours of age with a national coverage of >99% live births. Samples are posted for analysis to the laboratory in Auckland, which is the single facility conducting all newborn TSH tests in the country. The Perkin–Elmer AutoDelfia assay was used during the period of the study to measure whole‐blood TSH. Delfia is a solid-phase, two-site immunofluroassay and is based on the direct sandwich technique in which two monoclonal antibodies are directed against two separate antigenic determinants on the TSH molecule. It measures the whole blood and hence has a value approximately 2.2 times that of plasma in a 2-day-old neonate. In the NMSP, there was a change in assay platform from AutoDelfia (AD) to genetic screening processor (GSP) in March 2019, but no significant change in the normal TSH level was noted. TSH levels <15 mIU/L are considered normal by the NMSP [30].

## 3. Outcome

The outcome of the study was the incidence of permanent or transient thyroid dysfunction at birth in the offspring conceived within six months to mothers who had preconceptional OSCM HSG.

## 4. Results

Table 2 shows the TSH distribution of newborns whose TSH data were analysed. As described earlier in Table 1, the group comprised 146 consecutive traceable newborn babies of women who had undergone OSCM HSG in the Auckland region of New Zealand that were not of very low birth weight. This group included 79 female and 67 male infants with birth weights ranging from 1710 to 4660 gm. TSH levels were compared to 27,071 normal screens received from 1 January to 30 June 2020 by the NMSP (after excluding the screening cards from NICU babies, so that the TSH levels are more comparable with the neonates included).

We further grouped these neonates based on the proximity of their conception to OSCM HSG to analyse the effect of presumed higher iodine exposure in those conceived in the immediate months following the HSG.  Table 3 shows the distribution of TSH (mean, SD, median, and range) within each group of neonates conceived in a particular time frame following the OSCM HSG.

These data show that all babies had normal TSH levels (well below the cutoff, 15 mIU/L) on the newborn screening. The TSH ranged from 1 to 7 mIU/L on the whole blood analysis of DBS using the Perkin–Elmer AutoDelfia assay. The average TSH remained similar irrespective of the time elapsed from HSG to the conception. In addition, the 22 babies of the cohort excluded from analysis also had normal TSH at screening.

## 5. Discussion

In this retrospective cohort study, we did not identify any cases of CH among 146 newborns conceived following an OSCM HSG over the last 20 years nor did we identify a difference in TSH levels at newborn screening. 76% of these neonates were conceived in the immediate 3 cycles following OSCM HSG to their mothers, and in this group, contrary to our expectation from the previous Japanese data [25], we did not find a higher TSH compared to those conceived in the later cycles.

Table 4 compares our data with previous studies on the effect of preconceptional OSCM HSG on newborn thyroid function.

OSCM HSG using the standard Auckland approach [27] did not increase the risk for newborn thyroid dysfunction. These data are consistent with those from the Netherlands [28] and China [29, 31] but not with the data from Japan [25]. There were methodological differences in the approach to HSG between these groups, with larger OSCM volumes being used in the Japanese study among those who developed neonatal thyroid dysfunction. The Japanese are an iodine-sufficient population with some individuals having substantial iodine excess, probably related to their dietary differences. The New Zealand population is relatively iodine-deficient [32–34], and this might provide some protection from iodine excess; however, this explanation cannot solely explain our findings because the H2Oil offspring study [28] was conducted in the Netherlands, and the Dutch population is more iodine-sufficient [35]. Finally, it is also possible that the iodine excess secondary to OSCM occurs in the first two trimesters of pregnancy and reduces by the last trimester, so there is negligible impact on the neonatal thyroid gland by the Wolf–Chaikoff effect. This contrasts with the cases of congenital hypothyroidism following amiodarone use, dietary, or topical sources where high iodine excess continued until delivery.

The strengths of this research are a relatively large sample size and the uniformity of sample analysis. In addition, a TSH-based screening programme was used that identifies transient TSH elevations. The limitations are that potentially relevant information such as OSCM volume and maternal TSH levels were not recorded as this was a retrospective study. Preconceptional OSCM HSG can cause maternal thyroid dysfunction, particularly subclinical hypothyroidism (SCH) as shown in some studies [29, 36, 37]. As the fetus is entirely dependent on the placental transfer of maternal T4 in the first trimester, thyroid dysfunction in the mother during pregnancy including SCH could cause fetal hypothyroidism and subsequently influence the neurocognition of the offspring. Another point to note is that the newborn screening assay is not well calibrated at the low end as its purpose is to detect high levels >15 mIU/l blood. Therefore, low measurements are not very precise, and there may still be subtle differences in TSH levels that we were unable to demonstrate.

Overall, the results are reassuring, especially for the 90 children conceived in the immediate two cycles following the HSG. Thus, the results of our retrospective analysis suggest that OSCM HSGs do not cause a significant rise in the incidence of neonatal thyroid dysfunction. Further prospective studies are necessary to understand if maternal iodine levels and thyroid function following OSCM HSG influence the offspring's thyroid status at birth and their long-term neurocognitive outcome.

## Figures and Tables

**Table 1:** Baseline characteristics of newborns conceived following OSCM HSG along with population statistics of normal births in New Zealand.

		Neonates included	Neonates excluded	Normal births (01/01/2020–30/06/2020)
*n*		146	22	29573
Gender (male/female)		67/79	12/10	14989/14312^a^
Birthweight *n* (%)	<1000 g	0	3 (13.6)	295 (1.0)
	1000–1999 g	2 (1.4)	0	673 (2.4)
	2000–2499 g	7 (4.8)	2 (9.1)	1421 (5.0)
	>=2500 g	137 (93.8)	17 (77.3)	27183 (98.6)
Birthweight average (range)		3347 (1710–4660)	3061 (880–4090)	3372 (495–6090)
Multiple births		0	Twins 1 set	Not known

^a^Numbers may not add to the total as not all demographics are complete.

**Table 2:** TSH distribution of the neonates included compared to healthy neonates.

TSH mIU/L blood	Neonates included		Normal births (01/01/2020–30/06/2020)		*P* value
	*n*	%	*n*	%	
1	65	44.5	11,360	41.9	0.53
2	43	29.4	7,917	29.2	0.96
3	21	14.4	4,129	15.2	0.77
4	12	8.2	1,806	6.7	0.45
5	1	0.7	934	3.4	0.06
6	2	1.4	431	1.6	0.83
7	2	1.4	224	0.8	0.47
8	0		149	0.6	
9	0		75	0.3	
10–14	0		14	0.1	
>=15	0		32	0.2	
Total	146		27,071		

TSH: thyroid-stimulating hormone.

**Table 3:** TSH distribution of neonates conceived within various time frames after the OSCM HSG.

Time gap between HSG and conception	Number of neonates conceived within this time frame	Average TSH within the group mean (SD)	Median TSH within the group median (range)
1 month	45	1.9 (1.3)	2 (1–7)
2 months	45	1.9 (1.3)	2 (1–7)
3 months	21	1.9 (0.9)	2 (1–4)
4 months	11	2.2 (1.0)	2 (1–4)
5 months	16	2.5 (1.5)	2 (1–6)
6 months	8	1.8 (1.0)	1 (1–4)

TSH: thyroid-stimulating hormone.

**Table 4:** Comparison of our study with previous studies assessing the effect of OSCM HSG on the newborn's thyroid.

Study reference	Type of study	Country	Number of newborns with thyroid dysfunction	Volume of OSCM (Lipiodol) used during mother's HSG	Newborn screening strategy	Number of months between OSCM exposure and conception
Satoh et al. (2015) [25]	RS^a^	Japan	5 out of 212 OSCM HSGs	10 ml, 20 ml, 20 ml, NR, and NR	TSH-based screening	1, 1, 2, 3, and 12

Li et al. (2018) [29]	PS^b^	China	None out of 36 OSCM HSGs	NR^c^	TSH-based screening at 72+ hours	NR

Van Welie et al. (2020) [28]	RS	The Netherlands	None out of 76 OSCM HSGs	9 ml (6–11.8)	T4 followed by TSH if necessary	2.3 (1.1–4.3)

Our study	RS	New Zealand	None out of 146 OSCM HSGs	NR	TSH-based screening at 48+ hours	0–6
						Median 2
						IQR (1–3)

^a^RS: retrospective study; ^b^PS: prospective study; ^c^NR: not recorded; OSCM: oil-soluble contrast medium; HSG: hysterosalpingography; TSH: thyroid-stimulating hormone; T4: tetraiodothyronine.

## Data Availability

The data that support the findings of this study are available from the corresponding author upon reasonable request.
